# Impact assessment of construction waste policy intensity on environmental efficiency based on system generalized method of moments

**DOI:** 10.1007/s11356-024-32581-x

**Published:** 2024-02-29

**Authors:** Zezhou Wu, Minghao Gao, Peiying Xie, Heng Li, Mingyang Jiang

**Affiliations:** 1 State Key Laboratory of Intelligent Geotechnics and Tunnelling, Shenzhen, 518060 China; 2 Key Laboratory for Resilient Infrastructures of Coastal Cities (Shenzhen University), Ministry of Education, Shenzhen, 518060 China; 3 Sino-Australia Joint Research Center in BIM and Smart Construction, Shenzhen University, Shenzhen, 518060 China; 4 Department of Building and Real Estate, The Hong Kong Polytechnic University, Kowloon, Hong Kong

**Keywords:** Construction waste policies, Environmental efficiency, Data envelopment analysis (DEA), System generalized method of moments (SYS-GMM)

## Abstract

With the acceleration of urbanization in recent years, China has witnessed large-scale construction across its provinces, generating massive amounts of construction waste that pose challenges to environmental protection and sustainable development. This study evaluated the impact of construction waste policy intensity on its environmental efficiency. Firstly, the content analysis method was used to analyze the construction waste policy text quantitatively. Second, this study constructed a slack-based measure (SBM) model based on data envelopment analysis (DEA), considering resource input and construction waste output to measure environmental efficiency. Finally, we built and tested an econometric model of how policies affect environmental efficiency using the system generalized method of moments (SYS-GMM). The findings indicate a non-linear U-shaped link between policy intensity and environmental efficiency. Among all five control variables, population density, urbanization level, and technological innovation enhance environmental efficiency, while economic development and highway density will lower it. This study advances the research on construction waste policies and offers some insights for the construction industry to pursue sustainable development.

## Introduction

Since the reform and opening up, China’s provinces have urbanized rapidly, and the scale and number of construction projects have grown, producing a large amount of construction waste (Duan et al. 2015; Wang et al. 2021; Zhang and Tan 2020). Managing construction and demolition (C&D) waste is critical to the sustainable development of the construction industry (Li et al. 2022b; Liu et al. 2020). In order to reduce the generation of C&D waste, the government needs to formulate a series of targeted policies. In addition, the effect of policy implementation needs to be measured. At the same time, there are significant differences in the construction waste policies of local governments and the corresponding environmental efficiency levels (Hua et al. 2022; Liu et al. 2022a; Lv et al. 2021). Therefore, it is essential to analyze the impact of the local government’s construction waste policy on the environmental efficiency of C&D waste.

In the past, in the field of engineering construction efficiency research considering environmental factors, the research object mainly was carbon dioxide (Wang and Salman 2022; Zaborova and Musorina 2022). However, in the actual production process, construction waste is also the most critical pollutant in construction projects. At the same time, relevant scholars at home and abroad have not focused their research on the impact of policy intensity on the environmental efficiency of generating C&D waste. Many existing studies are more focused on environmental and economic benefits. According to the deficiencies of existing research, this study first proposes a method to measure the intensity of construction waste policy from the policy text perspective. It calculates the construction waste policy texts of 30 Chinese provinces as of December 2020. At the same time, according to the DEA, the SBM model containing the undesirable output was used to calculate the environmental efficiency level of C&D waste in 30 Chinese provinces and cities from 2011 to 2020. Finally, relevant variables are selected and described according to the political, economic, social, and technological (PEST) analysis method and existing research. The dynamic panel model is established using the dynamic panel data, and the SYS-GMM estimates the model’s results. The impact of construction waste policy intensity on environmental efficiency is analyzed based on the estimated results.

The innovations of this study are mainly in the following two areas: (1) Previous research on environmental efficiency in engineering and construction mainly focused on carbon dioxide, neglecting construction waste. In the actual production process, construction waste is also the most critical pollutant in the construction project, dramatically affecting the environment and the green development of the construction industry. Therefore, this study focuses on construction waste and draws more pertinent conclusions. (2) Previous literature lacks research on the impact of policy intensity on its environmental efficiency, and a predominance of environmental and economic perspectives. Given this, based on the existing research, this study started from a new perspective. Using the SBM model with undesirable outputs, this study established the input–output index system to detect the environmental efficiency of C&D waste. Then, SYS-GMM in the dynamic panel model was used to analyze the impact of construction waste policy intensity on its environmental efficiency, which supplemented the existing studies.

The rest of this research is organized as follows: “Literature review” section conducts a literature review of studies related to construction waste and environmental efficiency; “Methodology” section measures the intensity of construction waste policies; “Results and discussion” section measures the environmental efficiency of the generation of construction waste; “Conclusions” section analyzes the impact of policy intensity on environmental efficiency, and summarizes the main conclusions of this research.

## Literature review

This study begins by examining and analyzing three aspects of construction waste: relevant policies, the economic benefits that it generates, and the extent to which it affects the environment. Although there is not much research on the environmental effectiveness of building waste produced by academics domestically and internationally, environmental effectiveness integrates the advantages of construction waste from an economic and environmental standpoint. There is more research on both economic and environmental aspects of construction waste at home and abroad, so this study combed and analyzed the research contents of these two pieces.

### Construction waste management

In policy-related research, Calvo et al. (2014) established a management system for construction waste by economic incentives based on rewards and punishments and analyzed how the government affects this behavior. However, this study only focused on the economic dimension and did not consider the environmental impact. Lin et al. (2020) explored the impact of environmental and carbon tax policies on increasing C&D waste recycling rates and reducing carbon emissions. The results of this study showed that relevant policies and enacted laws are effective means to increase the recovery rate of C&D waste.

Research on the economic benefits of construction waste has focused mainly on the recycling and treatment of demolition phases. Liu et al. (2022b) explored the economic benefits of construction waste recycling enterprises under the tax incentives of the Guangzhou Municipal Government by establishing a system dynamics model, and using MATLAB for simulation and numerical analysis. They have found that value-added corporate income tax can help construction waste recycling companies improve their economic efficiency to a certain extent. Islam et al. (2019) conducted a study to analyze the economic benefits of reusing construction waste in other countries. The results showed that by recycling concrete and brick wastes, economic benefits can be increased to some extent.

Aiming at the environmental benefits created by construction waste, Marzouk and Azab (2014) constructed a dynamic model for construction waste management. The results, analyzed by STELLA software, showed that the utilization of construction waste recycling can significantly contribute to the reduction of greenhouse gas obligations, energy consumption, and the use of landfills. Ibrahim (2016) discussed how the waste management of a construction project affects the environment from the sustainability perspective, and the results confirmed that recycling and reusing construction waste can enhance economic and environmental benefits. By comparing the environmental benefits of different construction waste management schemes in Hong Kong, Hossain et al. (2017), using a full life cycle assessment, concluded that off-site sorting and straight to a landfill can have significant environmental impacts, while on-site separation can achieve better environmental benefits.

### Environmental efficiency assessment

Scholars’ research on environmental effectiveness at home and abroad mainly focuses on environmental efficiency measurement and its affecting elements. Using DEA techniques, Chen et al. (2015) evaluated the environmental efficiency of 30 Chinese provinces from 2001 to 2010 and verified their hypotheses. Ustun (2015) assessed the environmental effectiveness of 81 governorates in Turkey in 2010 using the DEA technique. Based on every single model, an environmentally efficient map of Turkey was structured, and the risk areas were identified for the country. In addition, when dealing with the undesired relaxation problem, scholars mostly use the non-radial SBM method in DEA to measure environmental effectiveness. Guo et al. (2019) utilized DEA to assess the environmental efficiency of industries in western China from 2001 to 2015 using SBM with undesirable outputs. Taleb et al. (2022) proposed an ultra-efficient SBM-DEA approach to gauge the environmental effectiveness of 19 Korean ports.

Currently, many studies on the factors affecting environmental benefits have been conducted, and scholars also conduct research and analysis from different perspectives, so the selected variables are not the same. Using a spatial panel model, Shen and Peng (2021) analyzed how industrial agglomeration affects industrial environmental effectiveness in China. The results showed a U-shaped correlation between industrial agglomeration and China’s environmental effectiveness of industry. Lu et al. (2020) employed a meta-stochastic frontier analysis two-step estimation approach to estimate environmental efficiency and analyze the influence of mayoral characteristics on environmental efficiency. The results showed that the effect of the mayor’s term of office on environmental efficiency has an inverted U-shape.

### Application of SYS-GMM

Generalized method of moments (GMM) is a parameter estimation method that plays a vital role in non-linear data processing research. A number of econometric models are designated not by full distributive hypotheses but by moment conditions, such as dynamic plane-data models with unobserved individual effects and microeconomic models with rational expectations, which are typically evaluated with the GMM. However, when estimating regression parameters using panel data, unobserved heterogeneity may give rise to stochastic perturbations that violate the general rules, leading to coefficient bias (Xie et al. 2021). To solve this problem, a combination of instrumental variables and GMM is necessary (Arellano and Bond 1991). Arellano and Bover (1995) and Blundell and Bond (1998) took the lead in proposing the SYS-GMM. Roodman (2009) pointed out in his research that SYS-GMM can introduce more instrumental variables with finite samples while reducing the coefficient bias. Compared to other GMMs, SYS-GMM overcomes intra-individual stationary effects, heteroskedasticity, and autocorrelation problems; solves endogeneity; and improves regression accuracy.

SYS-GMM is mainly used to study and analyze the effect of one variable on another. Scholars usually use it to research the implications of various elements on environmental, ecological, and technological efficiency. Gok and Sodhi (2021) examined the influence of governance practices on the quality of the environment in 115 high-, medium-, and low-income nations from 2000 to 2015 using SYS-GMM. Li et al. (2022a) developed a dynamic SYS-GMM model to benchmark the technological innovation efficiency of the Chinese manufacturing sector. Abudureheman et al. (2022) used stochastic frontier analysis and SYS-GMM to assess the energy rebound effect for 30 provinces in China from 2001 to 2017. However, there are few studies concerning using SYS-GMM in construction. Wang et al. (2023) explored the association between environmental regulations, carbon intensity, and technical innovation in China’s construction industry by using a panel database set of the construction industry in 30 provinces from 2004 to 2018 and analyzing it in conjunction with SYS-GMM. Yoo and Kim (2015) used SYS-GMM to explore the dynamic relationship between the growth and profitability of small and middle-sized building firms facing long-term economic stagnation in South Korea.

### Comprehensive review

A summary of existing studies reveals that domestic and foreign scholars have conducted various studies on construction waste management and environmental efficiency from different perspectives, but there are still shortcomings. For example, studies on the impact of policies on implementing construction waste minimization are relatively scarce and lack quantitative analysis. At the same time, corresponding studies on the environmental benefits generated by construction waste and the impact of implementing government policies on the environmental benefits are also absent. Furthermore, the application of SYS-GMM in the construction field lacks systematic research.

To address the shortcomings of existing studies, this research first estimated the policy intensity of construction waste and calculated its environmental effectiveness using the SBM model. On this basis, SYS-GMM was employed to analyze the effect of construction waste policy intensity on environmental effectiveness.

## Methodology

### Content analysis

Content analysis mines and retrieves text information based on the text and its related features. It quantifies and processes text information by collecting and sorting feature words, which then serve as relevant data for research. This study collected construction waste policy documents and used content analysis to construct a formula for measuring the intensity of policy texts based on policy text type and content relevance. It then assigned values to qualitative text materials and measured the intensity of construction waste policy using the formula.

When accurately calculating the number of policy texts on each type of construction waste, taking into account the lag and effectiveness of policy implementation time, the quantity of construction waste policies studied in this paper is a synthesis of the number of policies implemented in each province in that year and the number of policies that were not released in the same year but are still being implemented. If there is a policy that has expired in the current year, judge whether it is effective according to the expiration date: if the validity date expires before July, it will not be included in the number of policies for that year; if the validity date is after July, the policy will be included in the policy of the current year.

1 $${Q}_{nt}\text{=}{\sum }_{1}^{t}{q}_{nt}\text{- }{\sum }_{1}^{t-1}\text{Number of expired policies - Number of expired policies before July of the }t\text{ year}$$

where *n* represents each province (*n* = 1, 2, …, 30), *t* represents the year (*t* = 2011, 2012, …, 2020), *q* represents the number of construction waste policies implemented by each province in that year, and *Q* refers to the cumulative number of effective construction waste policies in that year.

The construction waste policy intensity indicates the impact of the policy on the implementation object. This paper chooses to study the content’s relevance and the policy text’s type. According to the research of relevant scholars on the quantification of policy texts, and at the same time combined with the actual situation of the selected quantitative indicators, the method of assigning fraction is used to reduce errors. The specific calculation formula is as follows:

2 $$C{d}_{nt}=\sum ({P}_{i}\times {C}_{j}\times {Q}_{ij})$$

Among them, *n* represents each province (*n* = 1, 2, …, 30), and *t* represents the year (*t* = 2011, 2012, …, 2020). Construction waste intensity (Cd) is composed of policy text type and policy content relevance assignment. *P* represents the type of text to which the policy belongs; *C* represents the fraction of relevance to the policy content. To account for the possibility of provinces implementing the same type of policy simultaneously, *Q* is introduced, which denotes the number of policies, i.e., the total number of policies implemented by the same province under the *i* and *j* types of policy documents.

### DEA

Economics, management science, and operation research scholars often use DEA to study efficiency. Through a review of representative literature on environmental efficiency evaluation index systems, this study established an environmental efficiency evaluation system suited for China from three aspects: economic development, resource consumption, and environmental pollution. Unlike most studies, this study included resource consumption as an input indicator to examine the environmental benefits of construction waste in each province, based on the Cobb–Douglas production function that assumes labor and fixed capital inputs as the main determinants of economic development. In other words, the specific input indicators include labor input, capital input, and resource consumption. Among them, the labor input indicators include the number of construction employees (in1); the capital input indicators include the total asset investment amount (in2); and the resource consumption indicators include steel (in3), wood (in4), cement (in5), glass (in6), and aluminum (in7). Meanwhile, the total profit and tax (out) was chosen as the desirable output indicator, and the amount of construction waste generated in each province (badout1) was chosen as the undesirable output indicator. Finally, This study referred to the research ideas and methods of Liu et al. (2022c), which measured the environmental efficiency of C&D waste in 30 Chinese provinces.

### SYS-GMM model

SYS-GMM utilizes level and differential changes and can avoid endogeneity and other related problems effectively. In addition, SYS-GMM is divided into a one-step method and a two-step method according to different selections of weight matrices. The latter method is more effective in dealing with the problem in this study. Therefore, the two-step SYS-GMM method was chosen to estimate the model parameters in this study. According to the existing research, there are two tests to assess the validity of the SYS-GMM instrumental variables and estimation results: one is the autocorrelation test, which uses the second-order sequential correlation test AR (2) to test whether the disturbance terms have a serial correlation. If the result checked is that the disturbance term does not have a two-stage autoregressive serial correlation problem, then the estimation result of the model can be considered valid. The second is the over-identification constraint test, which uses the Hansen test to test whether the selected instrumental variables are effective. Therefore, in this study, the test method used is the Hansen test to test whether the selected instrumental variables are effective. Dynamic panel estimation requires an instrumental variable over-identification test (Hansen test), and the Arellano-Bond AR (1) and AR (2) tests for the serial correlation of the residual items. The main steps involved assessing the environmental efficiency of C&D waste generation, importing panel data into STATA 16.0 software with relevant variables, and using SYS-GMM to analyze how construction waste policy intensity affects the environmental efficiency of C&D waste generation.

In order to test the impact of construction waste policy intensity on the environmental efficiency of construction waste generation, this study constructed a dynamic panel model with the environmental efficiency of the generation of C&D waste as the explained variable and construction waste policy intensity as the explanatory variable as follows:

3 $$T{E}_{it}={\beta }_{0}+{\beta }_{1}T{E}_{it-1}+{\beta }_{2}P{I}_{it}+{\beta }_{3}P{I}_{it}{}^{2}+{\beta }_{4}{\text{ln}}{X}_{it}+{\mu }_{i}+{\varepsilon }_{it}$$

where $i$ stands for the province ($i$ = 1, 2, ……, 30), $t$ is year ($t$ = 2011, 2012, ……, 2020), ${\text{TE}}_{\text{it}}$ is the environmental efficiency of the generation of C&D waste, ${\text{TE}}_{\text{it-1}}$ is the environmental efficiency of the generation of C&D waste with a one-period lag, ${\text{PI}}_{\text{it}}$ denotes construction waste policy intensity, ${\text{X}}_{\text{it}}$ is the control variables, including the level of ED, PD, UL, FD, and TI, ${\mu }_{\text{i}}$ denotes the unobservable effect of provincial differences, and ${\varepsilon }_{\text{it}}$ denotes the random disturbance term. The right side of the equal sign of Eq. (3) contains the one-period lagged term of the explained variable, which may have endogeneity problems when used as an explanatory variable, thus making the regression results incorrect. To address this situation, the method used in this study is the SYS-GMM, which reduces the bias of the regression findings by controlling for the endogeneity issue (Arellano and Bover 1995; Blundell and Bond 1998).

## Results and discussion

### Calculation of construction waste policy intensity

Considering the availability and validity of the data, the policy documents were collected through the official website of the local provincial people’s government, the official website of the provincial housing and urban–rural development bureau, and the law database of Peking University. Valid policy texts were retained, and 337 policy documents from 30 Chinese provinces from 2011 to 2020 were finally screened.

When the content analysis method was used, the text information was summarized by counting the keywords in the article or the title, and this information was used as the data for further statistical analysis. When setting the title, this research divided it into policy, first-level, second-level, and third-level titles. Therefore, 337 policy texts were coded according to “text number—first-level title—second-level title—third-level title.” Before in-depth quantitative analysis of the content of the construction waste policy texts, the types of policy texts to be studied were divided into statutory documents, rules and regulations, and plans. Among them, the statutory documents category includes notice, objection, and decision; the rules and regulations category includes ordinance, provision, measures, and detailed rules and regulations; the plans category includes plan and program.

According to the above Eq. (1), this study has counted the policy volume of different types of construction waste policy texts in China from 2011 to 2020, and the statistical results are shown in Table 1.

**Table 1:** Number of construction waste policies by policy text type from 2011 to 2020

Year	Statutory documents	Rules and regulations	Plans	Total
2011	46	55	20	103
2012	49	61	4	114
2013	51	67	8	126
2014	52	71	9	132
2015	53	79	10	142
2016	57	87	10	154
2017	58	97	13	168
2018	69	102	16	187
2019	80	114	19	213
2020	100	121	24	245

When considering the assignment to different content and types of policy texts, different policies have different influences and binding forces on subjects in the implementation process. For example, when planning policies are long, the implementation subject will attach more importance to them. As for rules and regulations policies that contain specific implementation strategies, they have to guide solid significance, so the scores are higher in the scoring process, thus reflecting the importance of policies. In terms of the relationship between policy contents, the higher the level of titles related to construction waste, the stronger the local government’s willingness to manage construction waste. This is also closely related to the research subject, so such policies can also get higher scores, as shown in Table 2.

**Table 2:** Subdivision and assignment results of policy documents

Category	Policy subdivision	Assignment
Policy text type	Statutory documents	1/3
	Rules and regulations	1/2
	Plans	1
Relevance of policy content	Policy title	1
	First-level title	1/2
	Second-level title	1/3
	Third-level title	1/4

Regarding the relevance degree of policy content, assign 1, 1/2, 1/3, and 1/4 to the policy title, first-level title, second-level title, and third-level title, respectively. In addition, according to the degree of influence reflected in each category of policy texts, the assignments for statutory documents, rules and regulations, and plans are 1/3, 1/2, and 1, respectively. According to the above Eq. (2), the calculation results of the construction waste policy intensity of 30 provinces from 2011 to 2020 are shown in Table 3.

**Table 3:** Construction waste policy intensity values for 30 provinces in China from 2011 to 2020

Province	2011	2012	2013	2014	2015	2016	2017	2018	2019	2020	Mean
Beijing	2.28	5.20	6.53	66.20	7.62	8.12	8.62	11.45	14.71	17.30	14.80
Tianjin	1.51	1.51	1.51	2.83	3.08	3.08	4.08	4.73	4.73	6.12	3.32
Hebei	1.37	1.70	1.70	2.19	2.19	3.70	3.87	4.48	4.81	6.16	3.22
Shanxi	0.94	0.94	0.94	0.94	0.94	1.27	1.77	3.10	3.27	3.35	1.75
Inner Mongolia	0.00	0.11	0.11	0.11	0.11	0.11	0.11	0.11	0.53	0.53	0.18
Liaoning	0.75	0.75	1.09	1.09	1.09	1.09	1.43	1.09	1.09	1.26	1.07
Jilin	0.13	0.13	1.63	1.63	1.63	1.63	1.96	1.96	1.79	2.12	1.46
Heilongjiang	2.11	2.11	2.11	2.11	2.11	3.12	4.47	6.68	6.68	8.91	4.04
Shanghai	7.89	8.23	8.23	8.23	12.90	13.40	14.07	15.18	16.54	17.04	12.17
Jiangsu	2.04	2.71	2.71	2.71	3.21	3.54	3.71	4.05	4.05	4.05	3.28
Zhejiang	0.94	1.36	1.36	1.36	1.36	1.25	1.50	2.08	2.08	2.41	1.57
Anhui	1.26	1.26	1.26	1.26	2.02	2.02	2.02	2.78	2.78	3.14	1.98
Fujian	0.76	0.76	0.76	0.76	0.93	0.93	0.93	1.10	1.44	1.61	1.00
Jiangxi	0.08	0.08	0.08	0.08	0.08	0.08	1.33	1.33	1.33	1.33	0.58
Shandong	2.45	2.87	3.12	3.12	3.12	3.37	3.37	3.87	3.87	4.12	3.33
Henan	0.27	0.69	0.69	0.86	1.97	2.22	2.22	3.32	3.32	3.32	1.89
Hubei	0.97	0.97	0.97	0.97	0.97	1.31	1.64	1.64	2.23	2.34	1.40
Hunan	0.64	0.64	0.64	0.64	0.64	0.64	0.64	0.89	1.55	2.85	0.98
Guangdong	1.34	1.34	1.67	1.67	2.35	2.18	2.18	2.36	2.46	3.04	2.06
Guangxi	0.77	0.77	0.66	0.66	0.66	1.93	1.69	1.61	2.46	2.87	1.41
Hainan	2.01	1.34	1.34	1.34	1.34	1.34	1.84	1.84	2.34	2.84	1.76
Chongqing	2.22	2.11	3.10	3.09	2.59	2.59	2.94	4.20	4.64	6.09	3.36
Sichuan	0.87	1.12	1.12	1.37	1.37	1.37	1.37	2.54	3.05	3.05	1.72
Guizhou	0.59	0.93	0.93	0.93	1.26	2.04	2.37	2.54	2.54	5.50	1.96
Yunnan	0.25	1.19	1.19	1.19	1.30	1.30	1.30	1.30	1.58	1.69	1.23
Shaanxi	0.25	0.25	0.25	1.17	1.17	1.84	2.59	2.59	3.34	3.62	1.71
Gansu	0.36	0.36	0.36	0.36	0.36	0.36	0.36	0.69	1.17	0.92	0.53
Qinghai	0.00	0.00	0.00	0.00	0.00	0.11	0.11	0.61	0.95	1.12	0.29
Ningxia	0.16	0.49	1.65	1.65	1.65	1.65	2.49	2.49	2.99	2.99	1.82
Xinjiang	0.28	0.70	0.70	0.70	0.70	0.70	0.87	1.04	2.04	2.21	0.99

### Calculation of environmental efficiency value of construction waste

In this study, the SBM model was adopted to estimate the environmental efficiency of construction waste generated in 30 provinces of China. The panel data of 30 provinces were collected from China Statistical Yearbook and China Construction Industry Statistical Yearbook, spanning 10 years from 2011 to 2020. In addition, for the scientific consideration of the input and output indicators, this study also adopted the Pearson correlation test. It is mainly used to check whether the input and output indicators comply with the “homogeneity” principle. The results showed that at the 0.01 level of significance, there was a remarkable positive association between seven input indicators and two output indicators, which principally conforms to the “homogeneity” demanded by the DEA model, and the next step of the study could be conducted.

To evaluate the environmental efficiency produced by construction waste, this study establishes an SBM model considering undesirable outputs based on the relevant methods and research ideas mentioned in the “DEA” section and estimated the environmental efficiency generated by construction waste in 30 provinces in China from 2011 to 2020 using MaxDEA software. The computational results are shown in Table 4.

**Table 4:** Calculation results of environmental efficiency of construction waste

DMU	2011	2012	2013	2014	2015	2016	2017	2018	2019	2020	Mean
Beijing	1.00	1.00	1.00	1.00	1.00	1.00	1.00	1.00	1.00	1.00	1.00
Tianjin	1.00	1.00	1.00	1.00	1.00	0.44	1.00	1.00	1.00	0.36	0.88
Hebei	0.42	0.41	0.43	0.39	0.39	0.41	0.35	0.50	0.33	0.25	0.39
Shanghai	1.00	1.00	1.00	0.70	1.00	0.52	0.46	1.00	0.43	0.33	0.75
Jiangsu	1.00	1.00	1.00	1.00	1.00	1.00	1.00	1.00	1.00	1.00	1.00
Zhejiang	1.00	1.00	0.52	0.60	0.55	0.52	0.49	0.36	0.33	0.31	0.57
Fujian	0.76	0.76	0.55	0.56	1.00	1.00	1.00	1.00	1.00	1.00	0.86
Shandong	1.00	0.51	0.81	0.84	0.61	0.54	0.60	0.70	0.48	0.37	0.64
Guangdong	1.00	1.00	1.00	1.00	1.00	0.63	0.73	1.00	0.56	0.30	0.82
Hainan	1.00	1.00	1.00	1.00	1.00	1.00	1.00	1.00	1.00	1.00	1.00
Shanxi	0.64	1.00	0.62	0.66	0.58	0.40	0.39	0.67	0.53	0.34	0.58
Anhui	0.54	0.66	1.00	0.45	0.64	0.54	0.49	0.66	0.37	0.40	0.57
Jiangxi	0.37	1.00	1.00	1.00	1.00	0.62	0.53	0.58	0.50	0.54	0.72
Henan	1.00	1.00	1.00	1.00	0.80	1.00	1.00	1.00	1.00	1.00	0.98
Hubei	1.00	1.00	1.00	1.00	1.00	0.56	0.60	1.00	1.00	1.00	0.92
Hunan	1.00	1.00	1.00	1.00	1.00	0.55	0.50	1.00	0.50	0.67	0.82
Inner Mongolia	1.00	1.00	1.00	1.00	0.48	0.41	0.54	0.64	0.65	0.48	0.72
Guangxi	0.31	0.34	0.31	0.29	0.40	0.36	0.33	0.42	0.41	0.30	0.35
Chongqing	0.69	1.00	0.84	1.00	1.00	1.00	1.00	1.00	0.86	1.00	0.94
Sichuan	0.47	0.47	0.45	0.35	0.37	0.27	0.30	0.63	0.40	0.48	0.42
Guizhou	0.28	0.26	0.24	0.25	0.20	0.15	0.30	0.48	0.31	0.26	0.27
Yunnan	0.64	1.00	1.00	0.54	0.74	0.59	1.00	1.00	1.00	1.00	0.85
Shaanxi	1.00	1.00	0.76	0.65	0.50	0.46	0.44	0.66	0.41	0.34	0.62
Gansu	0.41	0.52	0.49	0.50	1.00	0.60	0.75	0.86	0.80	0.30	0.62
Qinghai	1.00	1.00	1.00	1.00	1.00	1.00	1.00	1.00	1.00	1.00	1.00
Ningxia	1.00	0.68	0.43	1.00	0.56	1.00	1.00	1.00	1.00	1.00	0.87
Xinjiang	0.31	0.55	1.00	1.00	1.00	1.00	1.00	1.00	1.00	0.37	0.82
Liaoning	1.00	1.00	1.00	0.49	0.51	0.26	0.35	1.00	0.36	0.26	0.62
Jilin	1.00	0.38	0.42	1.00	0.73	0.65	1.00	1.00	1.00	0.64	0.78
Heilongjiang	0.45	1.00	0.57	1.00	1.00	1.00	0.58	1.00	1.00	0.59	0.82

### Analysis of the influence of policy intensity on environmental efficiency

#### Theoretical basis and research assumptions

Based on different assumptions and theoretical frameworks, there are mainly two opposed hypotheses about the impact of environmental regulatory policies: the compliance cost hypothesis and the Porter hypothesis. The basic idea of the former is that environmental control policies will encourage enterprises to innovate while increasing their costs, thereby forming a technological innovation effect and improving environmental efficiency. Under the existing static analysis framework, the general assumption is that the technology and resource allocation remains unchanged, and the enterprise chooses the configuration method that minimizes the cost. Therefore, adopting the construction waste policy will increase companies’ costs and reduce waste generation, which may negatively impact the enterprises affected (Gray and Shadbegian 2003). Improving environmental quality is important for enterprises to implement environmental control policies. It must invest human, financial, and material resources and other policy implementation costs in this process. When funds are limited, environmental governance becomes more costly, which reduces enterprises’ production capacity and productivity. This leads to lower profits and less motivation for production, negatively affecting economic growth and environmental efficiency.

On the contrary, Porter and Vanderlinde (1995) believe that environmental regulation policies can stimulate independent innovation of enterprises and provide enterprises with competitiveness. Under the requirements of green development, the supervision of environmental control policies has been continuously strengthened, and the cost of enterprises has continued to rise. Companies will enhance their production procedures and support technical innovation to increase their competitiveness, lower costs, lower the cost of pollution, and maximize profits. At the same time, it also compensates for the environmental management costs caused by environmental regulation, improving the corresponding environmental efficiency.

In addition, environmental control policies can also have positive and negative effects through regulation by coercive means. When the policy was first implemented, the government’s management system was not in place, the relevant supervision was not fully implemented, and the whole system was still in the fumbling stage. Moreover, the government set a standard in such a situation. It did not consider the differences between enterprises, making it necessary to comply with such rules no matter which kind of enterprise, which tends to make enterprises form a rebellious mentality. Meanwhile, some companies may bribe regulators to protect their interests, resulting in poor policy enforcement, economic damage, and inefficient waste reduction. From a long-term perspective, the government’s management of policies is getting stronger, and the relevant system tends to be improved, so different policies can be formulated for the actual situation of different enterprises. If the policy intensity is set properly, reducing resource consumption and pollution emissions will be immediate and help improve environmental efficiency.

Because of this, this study proposed a hypothesis that the intensity of construction waste policy has a U-shape effect on the environmental efficiency of generating C&D waste by first inhibiting and then promoting.

#### Variable selection

In order to investigate how construction waste policy intensity affects the environmental efficiency of the generation of C&D waste, this study selects the environmental efficiency of the generation of C&D waste as the explained variable, and construction waste policy intensity as the explanatory variable. In addition to policies, other factors may influence the environmental efficiency of construction waste generation. It is necessary to introduce other factors that may impact the construction waste generation problem in the model to control for effects other than experimental variables. The PEST analysis and previous studies were the foundation for the variable selection. Among them, PEST analysis is a widely used environmental analysis method, mainly composed of four types of external environmental factors: political, economic, social, and technical. Based on this, GDP per capita, population density, urban population share, highway density, and number of patents granted were selected as control variables in this study, and the selected variables are specified as follows:

1. Explained variable
  - Environmental efficiency (EE): Environmental efficiency of the generation of C&D waste in 30 provinces in China from 2011 to 2020 was measured using the SBM model in the “DEA” section.
2. Explanatory variable
  - Policy intensity (PI): This section focuses on exploring the effects of construction waste policy intensity on construction waste environmental variables, so the construction waste policy intensity measured in the “Content analysis” section is chosen as the explanatory variable.
3. Control variables
  - Economic development (ED): The better the level of economic development in a province, the more it focuses on the environmental issues of the city, which can significantly enhance construction waste management in engineering projects, thus having a positive impact on the environmental efficiency of C&D waste generation. Therefore, the GDP per capita is used in this study to measure economic development.
  - Population density (PD): Population density can reflect a region’s economic development and land scarcity. Because construction waste will take up some land resources when dumped randomly, the areas that pay more attention to the construction waste generation problem may be those with less available land resources. Therefore, population density can be used to measure the environmental efficiency of generating C&D waste. Population density data are measured using the ratio of living population to land area.
  - Urbanization level (UL): In the process of urbanization development, a lot of construction waste will be generated, which poses a severe threat to the environment and puts tremendous pressure on the ecological environment. This study selects the proportion of the urban population to measure urbanization level.
  - Freeway density (FD): Freeway density indicates the region’s accessibility, and its higher level can lower the delivery and construction cost of construction waste, thus decreasing the amount of waste sent to landfills, which may positively affect the environmental efficiency of C&D waste generation. This research uses the ratio of standard freeway miles to land area to measure the freeway density index.
  - Technology innovation (TI): Scientific and technological innovation is the primary driver of technological progress, which helps improve economic development and is essential to improve the environmental efficiency of generating C&D waste. This study chooses the number of granted patent applications as the index to measure technological innovation.

#### Data sources and descriptive statistical analysis

This research used 30 provinces in China as the sample and collected panel data for the 10 years from 2011 to 2020. Data on GDP per capita, resident population, proportion of urban population, standard freeway mileage, and number of granted patent applications are from the annual China Statistical Yearbook from 2012 to 2021, and data on land area are from the China Administrative Region Network. The explained variable is the environmental efficiency value of the generation of C&D waste calculated by the SBM model with undesirable outputs in the “Calculation of environmental efficiency value of construction waste” section. On this basis, SPSS software was used to conduct econometric analysis, and the descriptive analysis results of relevant variables were obtained, as shown in Table 5.

**Table 5:** Results of descriptive analysis of variables

Variables	Number of observations	Mean	Standard deviation	Minimum value	Maximum value
EE	300.00	0.74	0.28	0.15	1.00
PI	300.00	2.56	4.61	0.00	66.20
ED (unit: billions of dollars)	300.00	56,385.68	27,306.47	16,413.00	164,889.00
PD (unit: persons/kilometer squared)	300.00	470.55	707.44	7.86	3949.21
UL (unit: percentage)	300.00	59.01	12.22	35.03	89.60
FD (unit: kilometers/hundred square kilometers)	300.00	319.04	267.30	8.76	1341.27
TI (unit: number of pieces)	300.00	58,602.22	89,366.55	502.00	709,725.00

From Table 5, the sample size for each variable is 300, but there is a large gap between each variable’s minimum and maximum values. Descriptive statistics can reflect the basic information of each variable, but further data analysis is still needed to explore the relationship between the variables in depth.

#### Model construction

In order to test the impact of construction waste policy intensity on the environmental efficiency of construction waste generation, this study constructed a dynamic panel model with the environmental efficiency of the generation of C&D waste as the explained variable and construction waste policy intensity as the explanatory variable.

In Eq. (3), the state of economic variables in a previous period may affect the next period. In order to better control for possible lagged effects, this study introduced first-order lagged terms of the explanatory variables to construct a dynamic panel model. To examine the non-linear effects of the explanatory variables, a quadratic term for the intensity of the construction waste policy was added to the model. In order to make the data smoother and to reduce the effects of multicollinearity and heteroskedasticity on the data to some extent, the relevant control variables were logarithmically processed.

#### Results and discussion

In this study, STATA 16.0 software was used to measure the impact of construction waste policy intensity on the environmental efficiency of construction waste generation, and the measured results are presented in Table 6. The results indicate that the *p*-value of AR (2) is found to be 0.566, which is greater than 0.1. The second-order serial correlation test AR (2) statistic is not significant, which means that there is no second-order serial correlation, which would also prove that the model is set up very reasonably and accurately. Additionally, the Hansen test’s *p*-value of 0.962 is higher than 0.1, indicating that all instrumental variables are exogenous and instrumental variables are valid.

**Table 6:** Results of estimating the impact of policy intensity on environmental efficiency

Variables	Coefficient	*T* value	*p*-value
L.EE	0.159	1.029	0.312
PI	− 0.041**	− 2.158	0.039
PI2	0.001**	2.170	0.038
lnED	− 0.033	− 0.149	0.883
lnPD	0.251**	2.496	0.018
lnUL	1.268*	1.990	0.056
lnFD	− 0.504***	− 3.170	0.004
lnTI	0.003	0.050	0.960
Constant term	− 2.736	− 1.635	0.113
AR (1)	0.025		
AR (2)	0.566		
Hansen	0.962		
Inflection point	20.5		

***, **, * represent significant at 1%, 5%, 10% levels, respectively

Based on the measured results of how construction waste policy intensity affects the environmental efficiency of C&D waste generation, it can be found that the coefficient of the lagged period of the environmental efficiency is not significantly positive, implying that the cumulative contribution of the environmental efficiency of C&D waste generation needs to be enhanced.

The results of estimating the effect of construction waste PI on the environmental efficiency of the generation of C&D waste are observed, and a U-type non-linear relationship between construction waste policy intensity and environmental efficiency of the generation of C&D waste is found, which passes the 5% significance test. The relationship between the two, first inhibiting and then promoting, suggests that the environmental efficiency of generating C&D waste can only be actively promoted once the policy intensity of construction waste crosses a certain inflection point. The point of inflection of the U-curve obtained through the calculation is 20.5. Only provinces with construction waste policy intensity higher than 20.5 can positively affect the environmental efficiency of the generation of C&D waste. If the construction waste policy is less intense than 20.5, then the policy in these provinces acts as a disincentive. Currently, the policy intensity of construction waste in all provinces is below the inflection point. Therefore, all provinces should vigorously strengthen the policy intensity of construction waste to cross the inflection point.

The effect of the level of ED on the environmental efficiency of the generation of C&D waste in the current period is negative and insignificant. According to the environmental Kuznets curve, an inverted U-shaped relationship exists between environmental pollution and economic growth (Kuznets 1955). When the economy was first developed, the waste of resources was particularly evident, leading to more serious environmental problems. The continuous development of the economy makes science and technology gradually play an increasingly important role, which also improves the quality of the environment. China’s construction industry had many policy advantages when it first developed. However, at that time, the lack of awareness of the whole industry in protecting the environment and the low threshold of environmental regulation leads to many environmental pollution problems. It seems that the growth of our per capita GDP may have come at the cost of the environment, as the higher level of economic development has reduced the environmental efficiency of our construction waste generation. Despite the growing attention to the minimization and recycling of construction waste in recent years, and the gradual transition of the construction industry to a green and sustainable development model, it will still require some time and effort.

PD is positive at a 5% significance level, and the environmental efficiency of construction waste generation increases by 0.251 units for every 1 unit increase in population density. It indicates that the environmental effectiveness of creating C&D waste is positively correlated with the growth in regional population density. It may be due to the scarcity of land resources in places with high population density and better infrastructure development, which can reduce pollutant emissions. The local people are relatively well educated, have a strong sense of environmental protection, and can consciously maintain the urban environment, thus helping to promote construction waste management and enhance the environmental efficiency of generating C&D waste.

UL is positively correlated with the EE of the generation of C&D waste in the current period at the 10% significance level. The environmental efficiency of the generation of C&D waste increases by 1.268 units for each unit increase in the population share of urbanization, indicating that the increase in the population share of urbanization contributes to the improvement of the environmental efficiency of the generation of C&D waste in China. In recent years, China has been advancing urbanization and incorporating the idea of ecological civilization into the entire process of urban development, reducing environmental pollution. The environmental efficiency of China’s construction waste generation and urban development have gradually achieved synergistic improvement.

The effect of FD on the environmental efficiency of the generation of C&D waste is significantly negative at the 1% level, and this negative effect deserves our attention. It may be because, in recent decades, China’s road infrastructure has become more and more perfect, and the number of engineering and construction projects in places with convenient transportation has gradually increased, producing an increasing amount of construction waste.

The coefficient of TI is not significantly positive, indicating that the environmental efficiency of the generation of C&D waste in China can be promoted through technological innovation. Technological innovation and development can reduce the amount of pollutants produced and improve the level of treatment of pollutants to protect the environment. At the same time, the transformation and application of scientific research results will have a demonstrative and learning effect on the whole industry, thus enhancing the level of environmental efficiency of the generation of C&D waste.

## Conclusions

To investigate the impact of construction waste policy intensity on the environmental efficiency of the generation of C&D waste, this study first quantified and measured provincial construction waste policies through content analysis. Second, the SBM model, which includes undesirable outputs, was used to construct a system of input–output indicators to measure the level of environmental efficiency of the generation of C&D waste. Finally, using the environmental efficiency of the generation of C&D waste as the explained variable and the policy intensity of construction waste as the explanatory variable, the SYS-GMM was applied to investigate the effect of policy intensity on the environmental efficiency of the generation of C&D waste. The specific findings are as follows.

The policy intensity of construction waste has a non-linear U-shaped relationship with the environmental efficiency of the generation of C&D waste, which is first inhibited and then promoted. That is, there is an inflection point before which policy intensity suppresses environmental efficiency. After crossing the inflection point, the environmental efficiency of each province will also gradually improve with the support of construction waste policies. In the current situation, the policy intensity of construction waste in each province is far less than the inflection point value, and the policy intensity of construction waste should be strengthened to cross the inflection point as quickly as possible. Besides, among the control variables, the effects of population density, urbanization level, and technological innovation on the environmental efficiency of generating C&D waste are positive. In contrast, the increase in economic development level and freeway density will reduce the level of environmental efficiency of the generation of C&D waste.

This study examines the impact of construction waste policy on the environmental efficiency of C&D waste generation, reveals its mechanism of action, and enriches the existing research on the field of construction waste policy. In addition, this study can provide some ideas for the sustainable and low-carbon development of the construction industry, as well as provide a theoretical basis for the state and relevant departments to promulgate some policies to protect the environment.

Of course, although this study has supplemented the existing relevant studies accordingly regarding research methods and ideas, it still has the following shortcomings due to data sources and time limitations. Firstly, this study adopts the content analysis method, which uses the most intuitive and scientific assignment criteria to quantify and measure the policy intensity of the construction waste policy directly from the type of policy text and the relevance of content. This study adopted the content analysis method to quantify and measure the policy intensity of construction waste policy directly from the policy text and content relevance using the most intuitive and scientific assignment criteria. However, in the current environment, this method has not been widely used in academia to measure policy intensity, and further research and exploration are needed to determine whether this method is more suitable for analyzing construction waste policies. Secondly, the selection of input–output indicators in this study is somewhat subjective, which may have overlooked the possibility of some potential elements that can be further explored in future research. Finally, when selecting the control variables of environmental efficiency of construction waste generation, five factors were selected through the PEST analysis method: economic development, population density, urbanization level, highway density, and scientific and technological innovation. There may be other factors that influence the explanatory variables, which can be improved in future research to achieve the influence factor analysis of comprehensiveness, scientificity, and rationality.

## Data Availability

Data will be made available on request.
